# Lack of Association of Estrogen Receptor Alpha Gene Polymorphisms with Cardiorespiratory and Metabolic Variables in Young Women

**DOI:** 10.3390/ijms131013691

**Published:** 2012-10-22

**Authors:** Ana Cristina Rebelo, Rozangela Verlengia, Vandeni Kunz, Nayara Tamburus, Alvaro Cerda, Rosario Hirata, Mario Hirata, Ester Silva

**Affiliations:** 1 Department of Morphology, (Campus II), Federal University of Goiás, 74001-970, Goiânia, GO, Brazil; 2 College of Health Sciences, Methodist University of Piracicaba, Rodovia Açucar, Km 151, 13400-911, Piracicaba, SP, Brazil; E-Mail: rverlengia@gmail.com; 3 Adventist University Center São Paulo, 13165-970, Engenheiro Coellho, SP, Brazil; E-Mail: vckunz@yahoo.com.br; 4 Federal University of São Carlos, Rodovia Washington Luís, km 235-SP-310, 13565-905, Sao Carlos, SP, Brazil; E-Mails: nayaratamburus@hotmail.com (N.T.); esilvas@unimep.br (E.S.); 5 Lab Applied Molecular Biology and Pharmacogenomics, Federal University of São Paulo, Av. Prof. Lineu Prestes, 58005508-900, Sao Paulo, SP, Brazil; E-Mails: tmalvarocerda@gmail.com (A.C.); rosariohirata@gmail.com (R.H.); mariohirata55@gmail.com (M.H.)

**Keywords:** estrogen receptor-α gene polymorphisms, heart rate variability, aerobic capacity, lipids

## Abstract

This study examined the association of estrogen receptor alpha gene (*ESR1*) polymorphisms with cardiorespiratory and metabolic parameters in young women. In total, 354 healthy women were selected for cardiopulmonary exercise testing and short-term heart rate (HR) variability (HRV) evaluation. The HRV analysis was determined by the temporal indices rMSSD (square root of the mean squared differences of successive R–R intervals (RRi) divided by the number of RRi minus one), SDNN (root mean square of differences from mean RRi, divided by the number of RRi) and power spectrum components by low frequency (LF), high frequency (HF) and LF/HF ratio. Blood samples were obtained for serum lipids, estradiol and DNA extraction. *ESR1* rs2234693 and rs9340799 polymorphisms were analyzed by PCR and fragment restriction analysis. HR and oxygen uptake (VO_2_) values did not differ between the *ESR1* polymorphisms with respect to autonomic modulation. We not find a relationship between *ESR1* T–A, T–G, C–A and C–G haplotypes and cardiorespiratory and metabolic variables. Multiple linear regression analysis demonstrated that VO_2_, total cholesterol and triglycerides influence HRV (*p* < 0.05). The results suggest that *ESR1* variants have no effect on cardiorespiratory and metabolic variables, while HRV indices are influenced by aerobic capacity and lipids in healthy women.

## 1. Introduction

Estrogens are important steroid hormones that influence multiple organ systems in both men and women, including cardiovascular, reproductive and skeletal muscle systems [1]. The effects of estrogens on the cardiovascular system are mediated mainly through the estrogen receptor (ER), which is a member of the nuclear hormone receptor superfamily and acts as a ligand-activated transcription factor [2,3]. Within the central nervous system, ER alpha is found in the preganglionic autonomic centers of the brain stem that are involved in cardiovascular regulation [4]. The autonomic nervous system plays a role in controlling the heart rate (HR) and vascular tonus, thereby helping to maintain homeostasis [3–5] and influencing cardiorespiratory capacity [6–9] and lipid profiles [10–12].

Association of the autonomic nervous system on the heart has been widely studied for the analyses of heart rate variability (HRV). HRV has previously been associated with aerobic capacity. Hedelin *et al.* [6] found that VO_2 peak_ is moderately and positively correlated with the high frequency band of HRV. This effect is attributed to parasympathetic modulation. In addition, Kouidi *et al.* [8] stated that autonomic adaptations of HR at rest are intrinsically linked with peak VO_2_. However, Loimaala *et al.* [9] claim that the highest HRV indices may not be linked to high aerobic capacity.

Metabolic variables, such as cholesterolemia, have been associated with low HRV. Previous studies have shown an inverse relationship between low HRV indices and high serum levels of total and LDL cholesterol in men with ischemic heart disease [10] and patients with coronary artery disease [11]. In addition, studies have shown that variations in plasma lipids depend on estrogen levels [13,14]. In women with augmented estrogen levels, Yildizhan *et al.* [14] observed both an increase in the levels of triglycerides and a reduction of HDL cholesterol in plasma. Estrogen receptor-mediated actions induce an increase in the metabolism of glucose and fat mass [15,16] and the regulation of peripheral vasodilation [2,15,17]. Both of these parameters are closely related to aerobic capacity [12].

The *ESR1* rs2234693 and rs9340799 polymorphisms have been reported as interfering with the action of the estrogen receptor [14–16], leading to the development of risk factors for cardiovascular disease, such as dyslipidemia, insulin resistance, hypertension, central obesity and type 2 diabetes [1,2,18–21]. Autonomic modulation of HR has been evaluated only in one study, which showed that the *ESR1* rs2234693 and rs9340799 polymorphisms in young healthy men are associated with a lower HRV, resulting from reduced parasympathetic autonomic modulation of HR [22]. On the other hand, the association of these genetic variants on the autonomic modulation of HR in women has not been described.

Although there is evidence that autonomic modulation of HR has been related to aerobic capacity [6,7], lipid profile parameters [10,11] and *ESR1* polymorphisms [2,18–22] in different sample populations, these associations in healthy young women have not been investigated. Therefore, this study investigated the relationship between the *ESR1* rs2234693 and rs9340799 polymorphisms and HRV, aerobic capacity and serum lipid profiles in young women.

## 2. Results and Discussion

The demographic characteristics, baseline cardiovascular data, functional aerobic classification and results of the blood and urine biochemical tests are shown in Table 1. These values are within the normal range for healthy young women.

Minor allele frequencies for *ESR1* c.454-397T > C and c.454-351A > G in the study group were 41.2% and 39.4%, respectively (Table 2). The genotype distributions were as expected from the Hardy-Weinberg equilibrium. A strong linkage disequilibrium was observed between the *ESR1* c.454- 351A > G and c.454-397T > C polymorphisms (Lewontin’s coefficient: D′ = 0.823; *p* = 0.001) and four haplotypes were detected in the sample. The most frequent haplotype (AT) was present in 59.2% of the studied chromosomes, whereas haplotypes GC, GT and AC had frequencies of 35.7%, 5.7% and 3.9%, respectively (Table 2).

The relationship between *ESR1* polymorphisms and cardiorespiratory and metabolic variables is shown in Table 3. The supine HRV indices in both the time domain (TD) and frequency domain (FD) (rMSSD, SDNN, LF, HF, LF/HF) were similar for all the genotypes. In addition, VO_2_ during peak CPET, which represents aerobic capacity, did not vary significantly between the genotypes investigated. Lipids (total cholesterol, triglycerides, LDL cholesterol and HDL cholesterol) levels were similar for the genotypes of both *ESR1* polymorphisms (c.454-397T > C and c.454-351A > G).

The present study demonstrated that these *ESR1* variants have no influence on cardiorespiratory and metabolic variables in healthy young women. A linear regression was performed to show that functional aerobic capacity and serum lipids had more of a direct association with HRV than genetic changes. Similarly, no association was found between the *ESR1* haplotypes and cardiorespiratory and metabolic variables (data not shown). Multiple linear regression analysis was used to evaluate the influence of peak VO_2_, HDL cholesterol, LDL cholesterol and triglycerides on HRV indices (Table 4). VO_2_ peak was positively related with rMSSD, SDNN and HF and negatively correlated with LF (*p* < 0.05). Triglycerides and total cholesterol were negatively correlated with rMSSD, SDNN, LF and HF, indicating that a relationship exists between these parameters and the autonomic modulation of responses. All the models built satisfied the hypotheses of homoscedasticity and normality of residuals.

Our results show a lack of association between *ESR1* polymorphisms (rs2234693 and rs9340799) and HRV indices analyzed in the time and frequency domains. These findings contrast those of Matsunaga *et al.* [22], who reported that these *ESR1* polymorphisms are associated with reduced autonomic control of HR in Japanese men in the time and frequency domains. This association could be a predictor for episodes of cardiovascular disease. Differences regarding the methods of signal processing and analysis of heart rate variability, type of experimental design and sample composition may have contributed to the different results obtained between Matsunaga’s study and ours. Matsunaga *et al.* [22] evaluated only young Japanese males who underwent ECG recording and power spectral analysis of HRV in the standing and supine position; however, the present study evaluated healthy young women in the supine position. Therefore, the common mechanisms of the molecular relationship between *ESR1* polymorphisms and the autonomic modulation of HR in healthy young women should be further explored. These results do not exclude the hypothesis that *ESR1* variants may contribute to the mechanism involved in the modulation of HR, but large-scale studies in other populations are needed to elucidate the influence of the *ESR1* rs2234693 and rs9340799 polymorphisms on the autonomic modulation of HR phenotypes.

In the present study, no relationship was found between *ESR1* genotypes and the serum lipid profile. Some studies have evaluated the impact of *ESR1* polymorphisms (rs2234693 and rs9340799) on basal serum lipids in patients with coronary artery disease (CAD) [2,21,23], including healthy females at peak reproductive age [24,25], postmenopausal females [20,26] and premenopausal female smokers [27]. In Iranian population with symptoms related to CAD subgroups of patients stratified by gender, Boroumand *et al.* [2] observed no effects of *ESR1* c.454-39T > C and c.454-351A > G variants on serum lipids and lipoprotein(a) levels. On the other hand, Molvarec *et al.* [24] reported that the total cholesterol concentrations in serum samples were significantly higher in healthy women carrying the *ESR1* c.454-39CC genotype than in those with the TT or TC genotypes. Whereas healthy women carriers of the c.454-351GG genotype had significantly higher total cholesterol and LDL cholesterol levels in serum samples compared to those with the AA or AG genotype in healthy Caucasian women and men of reproductive age. In this study, no differences were found in LDL, HDL, total cholesterol and triglyceride levels among carriers of genotypes for *ESR1* c.454-39T > C and c.454-351A > G variants, indicating that the relationship between the polymorphisms and lipid profiles of the mentioned studies depends on parameters of the experimental model, such as medication, gender, age and risk factors for CAD. Intronic polymorphisms are also known to modify the splicing of messenger RNA (mRNA) transcripts, resulting in significant changes in gene function. However, how the molecular mechanism of the C allele is associated with augmented estrogen action with respect to HDL cholesterol remains unclear. The single-nucleotide polymorphisms (SNPs) may be merely linked to another as-yet-unidentified causative sequence variant.

We also do not found any relationship between the *ESR1* c.454-39T > C and c.454-351A > G polymorphisms and peak VO_2_ during CPET and HRV at rest. The interaction of estrogen with ER alpha promotes peripheral vasodilatation, an effect that can contribute to an increase in functional aerobic capacity at peak effort. However, Gurd *et al.* [28] suggest that estrogen metabolism does not interfere with O_2_ uptake by muscle, as determined by deoxyhemoglobin/myoglobin (Delta HHb) values. Campbell *et al.* [29] found that postmenopausal women using estrogen hormone replacement therapy did not show an improvement in VO_2_ during physical training and that their estradiol levels remained unchanged. Molvarec *et al.* [24] demonstrated that healthy subjects at peak reproductive age who were carriers of the T and/or A alleles of *ERS1* SNPs (the recessive inheritance model) had higher estradiol levels, which is a protective factor against cardiovascular disease, as estrogen promotes ERα-mediated peripheral vasodilatation. Despite the cross-sectional nature and the sample size evaluated, some parameters were not controlled, which may have influenced the results of the study. In contrast, our study subjects were composed of only young healthy women who showed no change in aerobic capacity at peak VO_2_. When compared with other studies, the difference in the results may also be explained by the age of the participants and the experimental design.

Reduction of HRV, changes in the metabolism of plasma lipids and reduction in functional aerobic capacity are important risk factors for the development of CAD [11]. However, few studies have observed the relationship between HRV indices and healthy young women’s lipid profiles and aerobic capacities [10,12]. Thus, in the present study, linear regression revealed a significant correlation between VO_2_ during peak exercise and HRV indices. This finding suggests that increases in aerobic capacity are related to central adaptations and the autonomic modulation of HR. Kouidi *et al.* [8] found that autonomic modulation of HR at rest are intrinsically linked to functional aerobic capacity, which depends on the individual’s physical condition. However, according to Loimaala *et al.* [9], higher HRV indices may not be related to VO_2_, but rather may be related to microcirculation in the autonomic nervous system. The results suggest a relationship exists between resting HRV and functional aerobic capacity. These findings corroborate those of Hedelin *et al.* [6], Hautala *et al.* [7] and Aubert *et al.*, [30] all of whom reported that peak VO_2_ has a moderate positive correlation with the HF range, which suggests parasympathetic modulation. These authors also report that the lower values of BF correspond to central and peripheral adaptations, indicating that the reduction in sympathetic modulation is associated with gains in muscle performance, muscle blood flow and VO_2_ peak. Thus, the results suggest a relationship between the dynamics of HRV at rest with functional aerobic capacity.

We observed a significant and negative correlation between triglycerides and indices of autonomic modulation of HR (rMSSD, SDNN and HF). Other authors have observed an inverse relationship between HRV indices and total cholesterol and LDL values, both in patients with CAD [10,11] and in healthy young women [12]. In a study by Christensen *et al.* [10], men with CAD and healthy sedentary men were evaluated for the association between HRV indices and cholesterol. In both groups, total cholesterol and LDL were inversely associated with indices of HRV, *i.e.*, low levels of HRV are associated with high cholesterol levels. Researchers investigating the association between short-term HRV and cholesterol levels in both genders without heart disease found that the rMSSD was inversely related to LDL cholesterol [10–12]. Even though vagal tone (baroreflex sensitivity) and HRV have been shown to be reduced in individuals with a family history of dyslipidemia [11], the mechanism by which circulating lipids association HRV remains to be elucidated. Risk factors including a lack of physical activity and the abuse of tobacco, alcohol and drugs have also been associated with changes in lipid profiles, autonomic imbalance and decreased parasympathetic modulation [11]. On the other hand, regular physical training promoted the effective regulation of the autonomic nervous system, promoting an increase in parasympathetic modulation and a reduction of sympathetic modulation [31].

In order to maximize the vascular benefits on blood vessels in women with postmenopausal CVD, the potential interaction of estrogen with progesterone and testosterone and its effects on vascular function may need to be considered [31]. In summary, the results of the present study suggest that VO_2_ may be associated with indices of autonomic modulation of HR (rMSSD, SDNN and HF). Thus, regular physical training promoted an increase in parasympathetic modulation and a reduction of sympathetic modulation.

## 3. Experimental Section

### 3.1. Subjects and Study Design

The sample size was calculated by establishing an error of 10% and a power of 80% and using the higher allele frequency of the studied polymorphism described by previous works evaluating a Brazilian population [20]. Four hundred and forty-five healthy women who were 18 to 38 years old and self-described non-Africans (www.ibge.gov.br/) were selected for the study after being recruited through an advertisement campaign in gyms and clubs. Forty-nine participants were excluded and fifteen refused to participate. Twenty-two did not meet the inclusion criteria, twelve were missing data on one or more of the variables of interest (or had incomplete genotyping). These individuals had regular menstrual cycles and ovulation, which was confirmed by a serum progesterone concentration above 4.0 mg/mL on the 21st day of the menstrual cycle. An evaluation form on daily habits, previous family history of existing pathologies, use of oral contraceptives (OCs) and physical activity level was completed. All subjects were in good health, and their biochemical parameters were within normal range. Subjects showing clinical evidence and/or biochemical signs of hyperandrogenism, cardiac or respiratory disease, hypertension (blood pressure ≥140/90 mmHg), diabetes mellitus, thromboembolic disease, thyroid diseases, stroke, depression, or problem drinking and smoking were excluded from the study. None of the subjects were taking sedatives, antihypertensives, antiarrhythmics or any other medications that could affect the autonomic control of HR. The study was approved by the Ethics Committee of the Methodist University of Piracicaba, SP, Brazil (protocol # 43/06). All participants provided written informed consent.

#### 3.1.1. Clinical and Biochemical Assessment

All subjects underwent a clinical examination between the 7th and 10th day after the first day of menstruation. The body mass index (BMI) for each subject was calculated after the weight and height were measured. Resting HR was measured with a 12-lead electrocardiogram (ECG), and a cardiopulmonary exercise test was conducted using cardiac auscultation. HR and blood pressure (BP) were measured after 5 min of rest in the supine and sitting positions by the Korotkoff auscultatory method, using a mercury-column sphygmomanometer (WanMed São Paulo, SP, Brazil) and a stethoscope (Littman, St. Paul, MN, USA). These measurements were repeated every two minutes after the initial measurements were made and during two separate visits to the laboratory. For biochemical measurements, venous blood samples were drawn after a 12-h overnight fast. Serum glucose was measured by the glucose oxidase method. Levels of total cholesterol, high density lipoprotein (HDL) cholesterol, triglycerides, estrogen, progesterone, urea, and creatinine were determined by enzymatic colorimetric assays (BioSystems Biotecnica kit, Barcelona, Spain). For triglyceride values that were less than 400 mg/dL, low density lipoprotein (LDL) cholesterol was estimated using Friedewald’s formula.

#### 3.1.2. Clinical and Biochemical Assessment

A cardiopulmonary exercise test was carried out on a cycle ergometer (Quinton Corival 400, Seattle, WA, USA) with increments of 20 to 25 W·min^−1^ up to physical exhaustion. Physical exhaustion corresponded to an inability to keep up the speed of 60 rpm, the occurrence of a limiting symptom or the occurrence of respiratory fatigue. Power output increases were determined for each subject according to the following formula: power output increase (W) = [(height − age) × 14] − [150 + (6 × body mass)]/100 [32]. Ventilatory and metabolic measurements were obtained on a breath-by-breath basis using a specific metabolic analyzer (CPX/D MedGraphics Breeze, St. Paul, MN, USA). Aerobic capacity was then evaluated using absolute VO_2_ (mL·kg^−1^·min^−1^), which was obtained at the peak of the exercise test.

### 3.2. Heart Rate Variability Analysis

ECG and HRV were recorded beat to beat on a one-channel heart monitor (MINISCOPE II Instramed, Porto Alegre, RS, Brazil) and processed with an analog-to-digital converter (Lab PC+, National Instruments, Co., Austin, TX, USA), which acted as an interface between the heart monitor and a microcomputer. The ECG signal was recorded in real time after the analog-to-digital conversion at a sampling rate of 500 Hz [33]. The interval between an R wave and the next R wave (RR) was analyzed using 256 consecutive heart beats from the most stable section. The HRV analysis was carried out using linear methods. In the time domain, the temporal indices rMSSD (the square root of the mean squared differences of successive RRi divided by the number of RRi minus one, expressed in ms) and SDNN (root mean square of differences from mean R–R interval, divided by the number of RRi of the period selected) was used. In the frequency domain, a nonparametric method involving fast Fourier transformation of the previously selected RRi was used. Using power spectrum components, very low frequency (VLF: 0.003 to 0.04 Hz), low frequency (LF: 0.04 to 0.15 Hz) and high frequency (HF: 0.15 to 0.4 Hz) signals were obtained, as was the ratio between absolute low frequency and high-frequency areas (LF/HF ratio). Normalization was carried out by dividing the absolute power of ms2 (LF or HF) by the total power spectrum, subtracting the VLF component and multiplying by 100. HF and LF bands represent the action of parasympathetic and predominantly sympathetic components of HR regulation, respectively. The data were analyzed in MATLAB 6.5 using the HRV analysis routine [34].

### 3.3. Genotyping

DNA was isolated from white blood cells using the salting-out procedure [35]. *ESR1* c.454-397T > C (IVS1-397T > C, rs2234693) and c.454-351A > G (IVS1-351A > G, rs9340799) polymorphisms were determined using polymerase chain reaction (PCR) and restriction fragment analysis as previously described [19,20]. PCR assays were carried out in a Biometra T Gradient (Whatman Biometra, Göttingen, Germany) using the following cycling program: one cycle at 94 °C for 1 min, 30 cycles at 94 °C for 1 min 30 s, 62 °C for 1 min, and 72 °C for 90 s and one cycle at 72 °C for 10 min. PCR products were treated with endonucleases *Pvu*II and *Xba*I (Invitrogen, São Paulo, SP, Brazil). Restriction fragments (*Pvu*II: 936 bp and 438 bp; *Xba*I: 981 bp and 396 bp) were analyzed by 1% agarose gel electrophoresis. Genotyping quality control was performed as described in detail elsewhere [36]. All genotypes were determined by two independent technicians and the results were entered in the database in duplicate. Ten percent of the samples were randomly reanalyzed.

### 3.4. Statistical Analysis

The allele frequencies and genotype distribution were estimated by gene counting. The Hardy-Weinberg equilibrium was assessed by the chi-square test using Arlequin v3.11 software, which uses an expectation-maximization algorithm. Linkage disequilibrium (LD) and haplotype frequencies were estimated using the Lewontin’s D′ coefficient from each pair of polymorphisms and Haploview 4.2 software. SPSS v.16 was used to compare frequencies among the groups, adjusted residuals and the power of the test. The association between genotype groups and levels of cardiorespiratory and metabolic variables was analyzed using analysis of variance (ANOVA) with LSD’s correction for multiple comparisons. Multiple linear regression analysis was used to investigate the relationship between HRV indices and clinically relevant covariants: the functional aerobic capacity and the lipid profile. A minimum coefficient of determination (*R*^2^) of 30% and a variation of <0.03 were considered.

## 4. Conclusions

In summary, this study demonstrates the lack of an association between the *ESR1* polymorphisms and HRV indices, aerobic capacity, serum estradiol, progesterone and lipid profiles. This finding demonstrated that these *ESR1* variants have no association on cardiorespiratory and metabolic variables in young healthy women and suggested that they may not be implicated in cardiovascular risk in young women. However, the results also suggested that functional aerobic capacity and serum lipids may have an association with HRV indices.

## Figures and Tables

**Table 1:** Demographic, clinical data and metabolic variables of young women (*n* = 354).

Demographic and clinical data		Metabolic variables	
Age, years	26 ± 4	Glucose, mg/dL	71 ± 9
Body mass, kg	72 ± 2	Urea, mg/dL	0.54 ± 0.1
Height, cm	68 ± 13	Creatinine, mg/dL	0.6 ± 0.5
BMI, kg/m^2^	21 ± 3	Total cholesterol, mg/dL	161.2 ± 18
HR supine, bpm	60 ± 5	LDL cholesterol, mg/dL	96.0 ± 21
HR sitting, bpm	74 ± 10	HDL cholesterol, mg/dL	45.8 ± 10
SBP supine, mmHg	110 ± 4	Triglycerides, mg/dL	73.0± 22
DBP supine, mmHg	75 ± 3	Progesterone, ng/mL	1.84 ± 6
SBP sitting, mmHg	115 ± 3	Estradiol, pg/mL	101.9 ± 56
DBP sitting, mmHg	72 ± 3	Aerobic classification AHA	Regular

Values are shown as the mean ± standard deviation; BMI: body mass index; HR: heart rate; SBP: systolic blood pressure; DBP: diastolic blood pressure; HDL: high density lipoprotein; LDL: low density lipoprotein; AHA: American Heart Association.

**Table 2:** Frequencies of *ESR1* polymorphisms in young women.

Polymorphisms	Genotypes			Alleles	
c.454-397T > C (rs2234693)	TT 40.7% (144)	TC 36.2% (128)	CC 23.1% (82)	T 58.8%	C 41.2%
c.454-351A > G (rs9340799)	AA 42.4% (150)	AG 36.4% (129)	GG 21.2% (75)	A 60.6%	G 39.4%
Haplotypes	A–T 59.2%	G–C 35.7%	G–T 5.7%	A–C 3.9%	

Number of individuals is in parentheses.

**Table 3:** Relationship of *ESR1* polymorphisms with HRV and VO_2_ indices and metabolic variables in young women.

Variables	c.454-397T > C genotypes				c.454-351A > G genotypes			

	TT (*n* = 144)	TC (*n* = 128)	CC (*n* = 82)	*p*	AA (*n* = 150)	AG (*n* = 129)	GG (*n* = 75)	*p*
HRV and VO_2_ indices								
rMSSD, ms^2^	53.79 ± 34.39	53.42 ± 29.37	53.40 ± 41.82	0.43	53.13 ± 33.70	51.92 ± 31.40	53.16 ± 45.00	0.27
SDNN, ms^2^	55.54 ± 25.27	54.78 ± 23.83	57.34 ± 35.57	0.23	55.88 ± 26.17	53.36 ± 24.88	54.93 ± 37.39	0.24
LF, nu	0.45 ± 0.17	0.43 ± 0.16	0.46 ± 0.16	0.25	0.45 ± 0.17	0.44 ± 0.16	0.44 ± 0.18	0.27
HF, nu	0.54 ± 0.17	0.56 ± 0.16	0.53 ± 0.16	0.19	0.54 ± 0.17	0.55 ± 0.17	0.55 ± 0.18	0.71
LF/HF ratio	1.08 ± 0.87	1.01 ± 0.94	1.12 ± 1.02	0.21	1.06 ± 0.86	1.03 ± 0.88	1.07 ± 1.08	0.33
VO_2_, mL·kg^−1^·min^−1^	26.28 ± 5.53	27.24 ± 6.23	26.46 ± 15.60	0.12	25.66 ± 5.57	26.86 ± 5.88	26.46 ± 5.35	0.43
Metabolic								
Total cholesterol, mg/dL	166 ± 32	165 ± 35	161.6 ± 39	0.12	164 ± 34	164 ± 35	164 ± 39	0.19
HDL cholesterol, mg/dL	44 ± 13	45 ± 12	42 ± 9	0.14	44 ± 11	44 ± 12	43 ± 10	0.27
LDL cholesterol, mg/dL	98 ± 32	102 ± 37	97 ± 40	0.22	98 ± 33	100 ± 35	100 ± 41	0.32
Triglycerides, mg/dL	88 ± 47	85 ± 36	89 ± 59	0.43	84 ± 45	88 ± 41	89 ± 61	0.27

Data are shown as the mean ± standard deviation and compared by an analysis of variance (ANOVA) rank test. HRV: Heart rate variability; VO_2_: Oxygen uptake during peak exercise; rMSSD: square root of the mean of the sum of the squares of differences between adjacent RRi divided by the number of RRi minus one, expressed in ms); SDNN: square root of the sum of the squares of differences of individual values compared to the mean value, divided by the number of RRi in a period; LF: low frequency; HF: high frequency; ms^2^: square milliseconds; nu: normalized units. HDL: high-density lipoprotein; LDL: low-density lipoprotein.

**Table 4:** Multiple linear regression analysis of variables (VO_2_ and lipids) that influence HRV indices.

HRV indices	Aerobic capacity VO_2_		Lipids								

			HDL cholesterol		LDL cholesterol		Total Cholesterol		Triglycerides		
					
	B	*p*	B	*p*	B	*p*	B	*p*	B	*p*	*R*^2^
rMSSD	1.40	0.003	0.24	0.200	−0.10	0.290	−0.18	0.004	−1.79	0.020	48%
SDNN	1.01	0.001	0.08	0.590	−0.10	0.240	−0.15	0.002	−0.19	0.010	30%
LF	−1.73	0.010	−2.96	0.040	−0.09	0.960	−0.06	0.13	0.39	0.840	34%
HF	19.18	0.004	2.10	0.280	−0.01	0.700	−0.06	0.03	−2.45	0.150	42%

rMSSD: square root of the mean of the sum of the squares of differences between adjacent RRi divided by the number of RRi minus one, expressed in ms; SDNN: square root of the sum of the squares of differences of individual values compared to the mean value, divided by the number of RRi in a period; LF: low frequency; HF: high frequency; VO_2_: oxygen uptake during peak exercise; B: regression coefficient; *R*^2^: determination coefficient.
